# Metabolic dysfunction-associated steatotic liver disease affects the development of hepatocellular carcinoma after sustained virologic response in chronic hepatitis C patients

**DOI:** 10.1007/s00535-025-02270-8

**Published:** 2025-07-07

**Authors:** Shintaro Yamasaki, Takashi Nakahara, Masataka Tsuge, Kenji Yamaoka, Yasutoshi Fujii, Shinsuke Uchikawa, Hatsue Fujino, Atsushi Ono, Eisuke Murakami, Tomokazu Kawaoka, Daiki Miki, Shiro Oka

**Affiliations:** 1 Department of Gastroenterology, Graduate School of Biomedical and Health Sciences, Hiroshima University Hospital, Hiroshima, Japan; 2 Department of Gastroenterology and Hepatology, Hiroshima Prefectural Hospital, 1-5-54 Ujina-Kanda, Minami-ku, Hiroshima, 734-8530 Japan; 3 Liver Center, Hiroshima University Hospital, 1-2-3 Kasumi, Minami-ku, Hiroshima, 734-8551 Japan

**Keywords:** Chronic hepatitis C, Sustained virologic response, Cardiometabolic risk factors, Metabolic dysfunction-associated steatotic liver disease, Hepatocellular carcinoma

## Abstract

**Background:**

Despite the high success rate of direct-acting antivirals (DAAs) in achieving sustained virologic response (SVR) in patients with chronic hepatitis C virus (HCV) infection, the risk of hepatocellular carcinoma (HCC) persists in some patients. Cardiometabolic factors, including type 2 diabetes mellitus (T2DM), have been reported as risk factors for de novo HCC after SVR. However, the impact of metabolic dysfunction-associated steatotic liver disease (MASLD) on HCC development after SVR, particularly in Japanese patients, remains unclear.

**Methods:**

A total of 512 HCV-infected patients who achieved SVR following DAA therapy were enrolled in this study. Clinical and laboratory data at 24 weeks after the end of therapy (SVR24) were assessed to determine the impact of MASLD on the development of HCC. Risk factors for HCC occurrence were analyzed using the Fine and Gray subdistribution hazard model.

**Results:**

During a median follow-up of 56 months, HCC developed in 33 patients (6.4%). Patients with MASLD at SVR24 had a significantly higher cumulative incidence of HCC than those without MASLD (*P* < 0.001). Multivariable analysis identified MASLD, age, male, albumin-bilirubin-platelets (aMAP) score, and FibroScan-AST (FAST) score at SVR24 as independent risk factors for HCC development. Both aMAP and FAST scores were positively correlated with the number of cardiometabolic risk factors.

**Conclusions:**

MASLD is a significant determinant of post-SVR HCC risk among Japanese patients. Risk stratification incorporating MASLD, aMAP, and FAST scores may contribute to the development of optimized, patient-tailored HCC surveillance strategies and improve long-term outcomes in the Japanese clinical setting.

**Supplementary Information:**

The online version contains supplementary material available at 10.1007/s00535-025-02270-8.

## Introduction

With the advent of direct-acting antivirals (DAAs), sustained virologic response (SVR) has become nearly universal for patients with chronic hepatitis C virus (HCV) infection [1]. The eradication of HCV not only significantly reduces the burden of liver-related complications but also plays a crucial role in lowering the risk of hepatocellular carcinoma (HCC). In addition, the safety and efficacy of DAA therapy have been extensively validated across diverse patient populations, including those with advanced liver disease [2]. Achieving SVR substantially decreases the likelihood of HCC development associated with liver cirrhosis and progressive liver disease, marking a pivotal achievement in HCV management [3]. Despite these advancements, a subset of patients remains at risk of developing HCC even after achieving SVR. Various opinions exist regarding the association between factors categorized under cardiometabolic risk factors (CMRFs) and hepatocarcinogenesis. Those with consensus as risk factors for post-SVR HCC include diabetes mellitus, advanced age, high alpha-fetoprotein (AFP) level, and advanced liver fibrosis [4–8]. These findings underscore the necessity of continued surveillance and individualized patient management strategies even after viral eradication. Recent studies have reported that patients with metabolic dysfunction-associated steatotic liver disease (MASLD) remain at increased risk of developing de novo HCC following HCV eradication with DAA therapy in the Taiwanese population [9]. This finding highlights the importance of incorporating metabolic factors into the long-term management of patients post-SVR. The concept of MASLD was introduced through a Delphi consensus involving multiple international societies to refine the classification of fatty liver diseases, distinguishing it from nonalcoholic fatty liver disease (NAFLD) and improving disease terminology [10]. The new nomenclature aims to encompass diverse etiologies of steatotic liver disease (SLD) while avoiding stigmatizing language. MASLD is defined as a subset of SLD characterized by the presence of at least one CMRF, such as obesity, diabetes mellitus, or hypertension, while excluding other overt underlying causes of liver disease. Under this revised classification system, HCV-related liver disease falls under the “Miscellaneous” category within the broader SLD framework. This updated taxonomy reflects an evolving understanding of liver disease pathogenesis and underscores the need for precision in diagnosis and treatment planning. Despite the increasing recognition of MASLD and its associated CMRFs, few studies have specifically investigated its impact on HCC risk. To the best of our knowledge, no reports in Japan have examined the association between MASLD and hepatocarcinogenesis in post-SVR patients.

In general, HCV eradication is expected to improve metabolic profiles and hepatic steatosis, yet the trajectory of CMRFs, SLD, and MASLD in the post-SVR period remains insufficiently understood. Furthermore, the interplay between these conditions and their potential effect on HCC risk poses a significant clinical challenge.

We leveraged data from our institution to assess the risk of de novo HCC in patients who achieved SVR, with a particular focus on MASLD. In addition, predictive models such as the age, male, albumin-bilirubin, platelets (aMAP) score and the FibroScan-AST (FAST) score have been demonstrated as valuable tools for estimating HCC risk, providing clinicians with critical guidance for risk stratification [11–13]. Through this study, we aimed to clarify the impact of MASLD on subsequent HCC risk after HCV eradication in Japanese patients, which has been scarcely reported to date. Clarifying the role of MASLD may help to identify high-risk individuals who would benefit from more intensive HCC surveillance following HCV eradication. Such risk stratification approaches are expected to contribute to the development of optimized, patient-tailored surveillance strategies and support the improvement of long-term outcomes in the Japanese setting.

## Patients and methods

### Study population

**Fig. 1:**
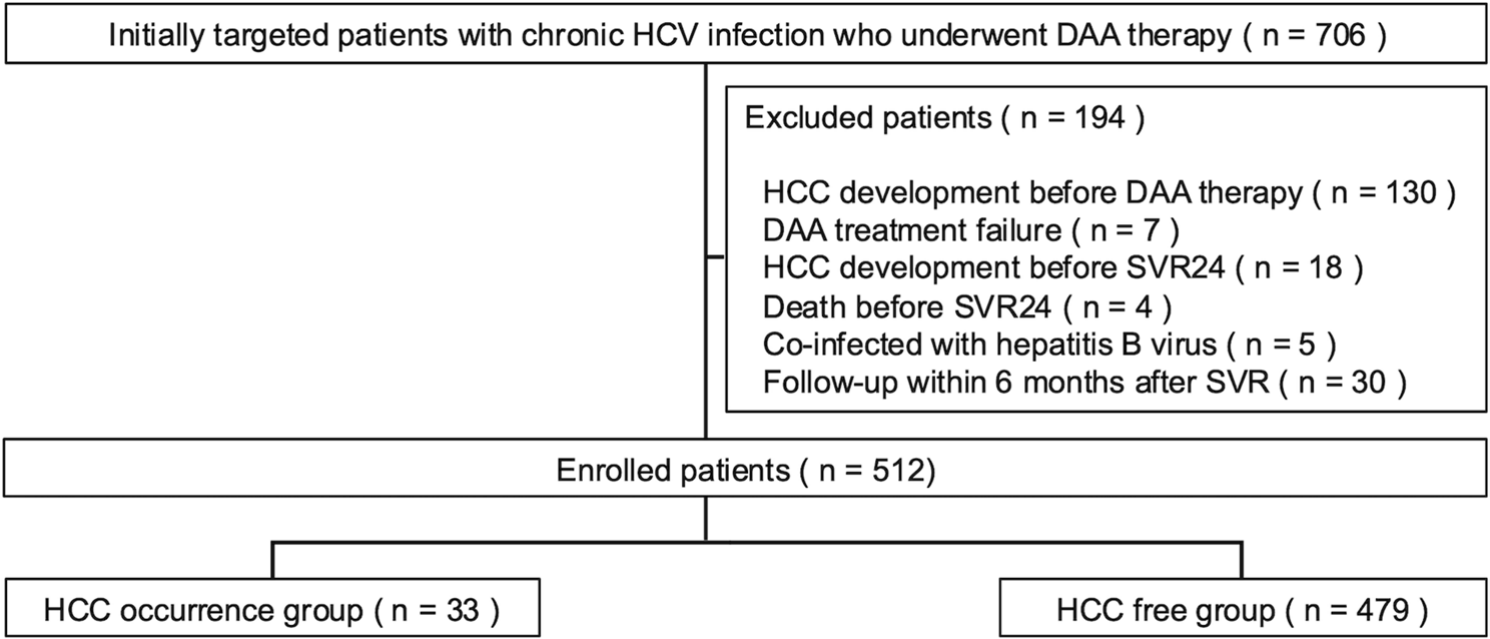
Patient enrollment procedures for the study. DAA, direct-acting antiviral; HCC, hepatocellular carcinoma; HCV, hepatitis C virus; SVR24, sustained virologic response 24 weeks after the end of treatment

A total of 706 consecutive patients with chronic HCV infection who received DAA therapy at Hiroshima University Hospital (Hiroshima, Japan) between April 2010 and March 2020 were retrospectively reviewed. The flowchart of the study population is shown in Fig. 1. Patients with a history of HCC before DAA treatment, DAA treatment failure, HCC development before 24 weeks after the end of therapy (SVR24), death before SVR24, co-infected with hepatitis B virus, and follow-up within 6 months after SVR24 were excluded from the study. Of the 706 patients who remained after applying exclusion criteria, 512 patients were enrolled in the study.

### Antiviral therapy

Patients were treated with DAAs, such as daclatasvir/asunaprevir for 24 weeks, ledipasvir/sofosbuvir for 12 weeks, ombitasvir/paritaprevir/ritonavir for 12 weeks, sofosbuvir/ribavirin for 12 weeks, or glecaprevir/pibrentasvir for 12 weeks.

### Clinical and laboratory assessments

Clinical and laboratory assessments were performed before treatment. HCV RNA levels were measured using the COBAS 135 TaqMan HCV test (Roche Diagnostics, Tokyo, Japan). The detection limit for the assay was 1.2 log IU/mL. Habitual alcohol consumption was defined as a weekly intake less than 140 g female, 210 g male (average daily less than 20 g female, 30 g male). CMRF was defined based on the cardiometabolic criteria of the MASLD diagnostic criteria, using the available data. CMRF includes five factors: (1) overweight + obesity, body mass index ≥ 23 kg/m^2^; (2) prediabetes + type 2 diabetes mellitus (T2DM), fasting serum glucose ≥ 100 mg/dL or glycosylated hemoglobin A1c (HbA1c) ≥ 5.7% or T2DM or on specific drug treatment; (3) hypertension, blood pressure ≥ 130/85 mmHg or specific drug treatment; (4) hypertriglyceridemia, plasma triglycerides ≥ 150 mg/dL, or specific drug treatment; and (5) hypo-high-density lipoprotein (HDL-C) cholesterolemia, plasma HDL-C ≤ 40 mg/dL for males and ≤ 50 mg/dL for females or specific drug treatment. Furthermore, steatotic liver disease was diagnosed using imaging studies at the SVR24 time point. SLD was diagnosed based on the presence of any of the following findings: positive hepatorenal echo contrast, positive hepatosplenic echo contrast, deep attenuation, or vascular blurring by abdominal ultrasonography, or liver-to-spleen attenuation ratio ≤ 0.90 or liver attenuation < 50 HU by computed tomography (CT). HCV-related MASLD (HCV-MASLD) was defined as the presence of SLD along with at least one of the CMRFs without habitual alcohol consumption at the SVR24 time point. The controlled attenuation parameter (CAP) and liver stiffness measure (LSM) were measured by FibroScan (Echosens, Paris, France). Considering the potential for measurement inaccuracies due to subcutaneous fat thickness when using the M-probe in obese patients, the XL-probe was preferentially used for patients with obesity. Patients were placed in the supine position with the right hand at maximal abduction for right lobe liver scanning. Measurements were considered valid when there were at least 10 measurements with LSM values of ≥ 60% and an interquartile range of < 30%, and the median value of these measurements was used for analysis. CAP and LSM data were not available in 164 patients (32.0%) because the measurements were not performed or failed due to technical reasons, such as obesity or limited patient compliance at the time of examination. Analyses regarding CAP and LSM were conducted based on the patients with available data. The fibrosis 4 index (FIB-4 index) was calculated using data for age, aminotransferase (AST), alanine aminotransferase (ALT), and platelet count according to the following equation: AST (U/L) × age/platelet count (× 10^9^/L) × ALT (U/L)^1/2^. The age–male–albumin–bilirubin–platelets (aMAP) score is a simple and objective risk model developed to predict the 5-year risk of HCC development in patients with chronic liver disease, irrespective of etiology and ethnicity [14]. The aMAP score was calculated using the following formula: aMAP score = ({0.06 × age + 0.89 × gender (male: 1, female: 0) + 0.48 × (log_10_ serum total bilirubin [μmol/L] × 0.66) + (serum albumin [g/L] ×  − 0.085) − 0.01 × platelet count (10^3^/mm^3^)} + 7.4)/14.77 × 100. The FibroScan-AST (FAST) score is a non-invasive algorithm developed to identify patients with non-alcoholic steatohepatitis (NASH) with significant activity (NAS ≥ 4) and advanced fibrosis (≥ F2), using LSM, CAP, and serum AST [15]. The FAST score was calculated using the following formula: FAST score = e^−1.65+1.07×In (LSM)+2.66*10–8×CAP3−63.3×AST−1^/1 + e^−1.65+1.07×In (LSM)+2·66*10–8×CAP3−63.3×AST−1^.

### HCC surveillance

After HCV eradication, all patients underwent HCC surveillance at least every 6 months using a combination of tumor markers, abdominal ultrasonography, and/or computed tomography (CT). HCC was diagnosed based on typical imaging findings, characterized by hypervascular enhancement in the arterial phase and washout in the portal or delayed phase. Diagnosis was confirmed by dynamic contrast-enhanced CT, magnetic resonance imaging (MRI), and/or angiography. In cases where lesions lacked characteristic enhancement, a fine-needle biopsy was performed for histological confirmation.

### Statistical analysis

Continuous variables were reported as median values with interquartile ranges. The cumulative incidence of HCC after SVR24 was estimated using the Kaplan–Meier method. For risk factor analyses of HCC development after SVR24, both univariable and multivariable analyses were performed using the Fine and Gray subdistribution hazard model to account for the competing risk of death. Continuous variables were analyzed as continuous variables without dichotomization to preserve statistical power. Serum markers with significantly skewed distributions, including AST, ALT, ɤ-GTP, AFP, and DCP, were log-transformed prior to analysis to improve distribution normality and model stability. Variables with a *P*-value of < 0.05 in the univariable analysis were included in the multivariable model. A *P*-value of < 0.05 was considered statistically significant in all analyses. All analyses were conducted using EZR (Saitama Medical Center, Jichi Medical University, Saitama, Japan), which is a graphical user interface for R (The R Foundation for Statistical Computing, Vienna, Austria). More precisely, it is a modified version of R commander designed to add statistical functions frequently used in biostatistics [16].

### Ethics

This study was conducted in accordance with the ethical principles outlined in the Declaration of Helsinki (revised in 1975) and was approved by the Ethics Review Committee of Hiroshima University (approval number: E-624–6). Informed consent was obtained from all participants through an opt-out approach, which was publicly disclosed on the hospital’s official website.

## Results

### Characteristics of enrolled patients

**Fig. 2:**
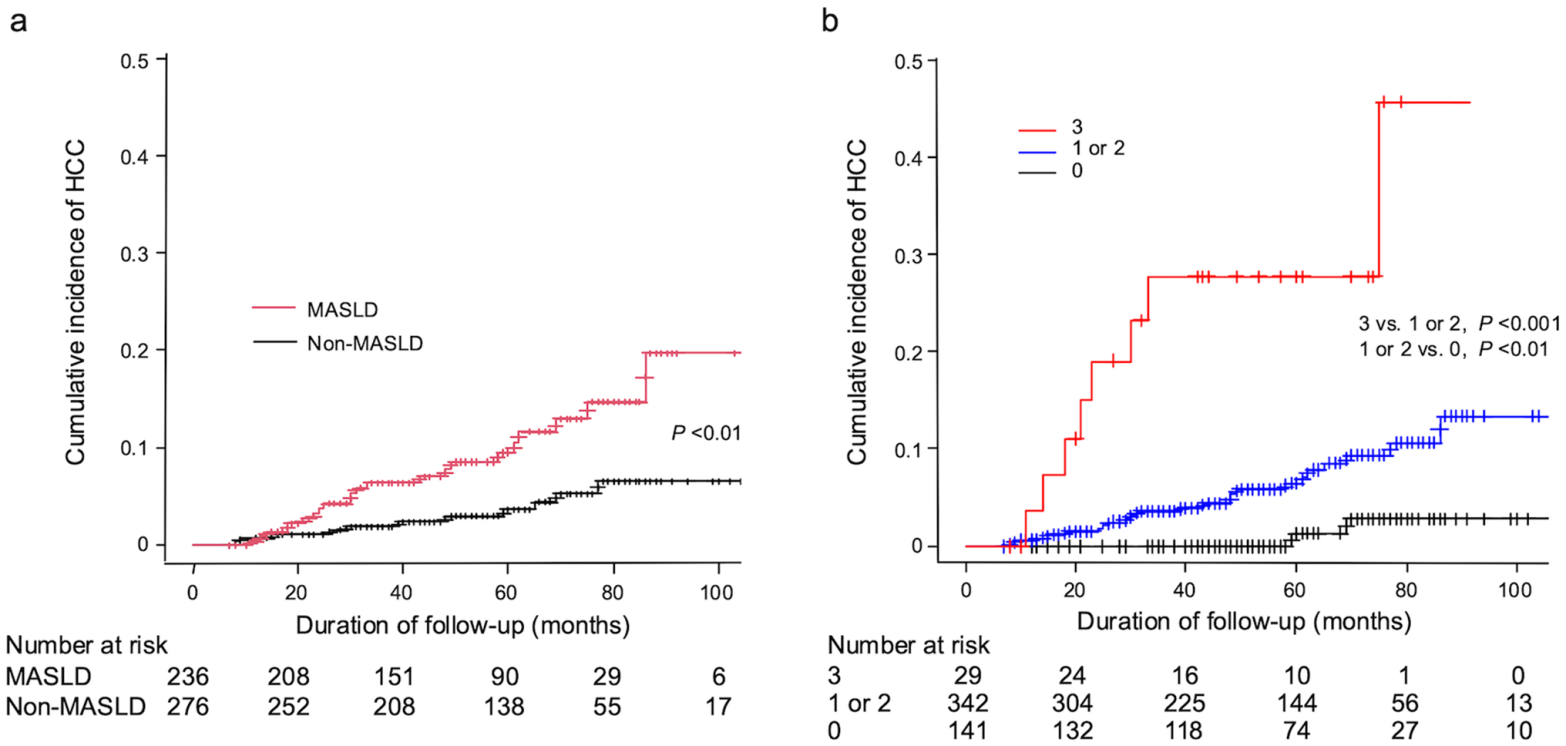
Cumulative incidences of hepatocellular carcinoma (HCC). **a** The cumulative incidence of HCC in patients with metabolic dysfunction-associated steatotic liver disease (MASLD; red line) was significantly higher than that in patients without MASLD (non-MASLD; black line) (*P* < 0.01). **b** Patients were divided into three groups according to the number of risk factors of HCC occurrence such as presence of MASLD, aMAP score ≥ 61 and FAST score ≥ 0.12 at sustained virological response 24 weeks after end of treatment. Patients having all three risk factors (red line), one or two risk factors (blue line), and with no risk factors (black line)

The clinical characteristics of the 512 patients are shown in Table 1. The median observation period after SVR24 was 56 (35–75) months. The median age of the participants was 65 years, and 213 individuals (42%) were male. MASLD was observed at SVR24 in 236 patients (46%). At the SVR24 time point, the median CAP was 211 dB/m, and LSM was 5.5 kPa. During the observation period, de novo HCC was developed in 33 (6.4%) patients. The annual incidence rate of HCC was 1.4% per year, and the cumulative incidence rate of HCC after SVR24 was 1%, 4%, 6%, and 10% at 1, 3, 5, and 7 years, respectively. Of the 236 patients with MASLD, 22 (9.3%) developed HCC, whereas of the 276 patients without MASLD, 11 (4%) developed HCC. The annual incidence rate of HCC was 2.2% per year for patients with MASLD and 0.8% per year for patients without MASLD. The cumulative incidence rate of HCC was significantly higher in patients with MASLD (3%, 6%, 10%, and 15% at 1, 3, 5, and 7 years, respectively) than in those without MASLD (1%, 2%, 4%, and 7% at 1, 3, 5, and 7 years, respectively) (subdistribution hazard ratio [SHR] 5.045, 95% CI: 2.200–11.57, *P* < 0.01) (Fig. 2a). In patients who developed HCC, not only the proportion of HCV-MASLD but also the prevalence of each CMRF component was higher than in HCC-free patients. Our cohort includes only 10 patients with metabolic and alcohol-associated liver disease (Met-ALD), whereas 3 patients (30.0%) developed HCC during the observation period. The annual incidence rate of HCC was 6.8% per year for patients with Met-ALD, which appears to be high.

**Table 1:** Characteristics of all patients at SVR 24 weeks after the end of treatment in a combined cohort

	All patients		
	Combined cohort (*n* = 512)	HCC occurrence (*n* = 33)	HCC-free (*n* = 479)
*Patient’s character*			
Age, years	65 (57–75)	68 (60–79)	67 (57–75)
Gender, male (%)	213 (42)	17 (52)	196 (41)
BMI, kg/m^2^	22 (20–25)	23 (21–25)	22 (20–25)
Smoker (%)	189 (37)	15 (45)	174 (36)
Habitual alcohol (%)	38 (7)	5 (15)	33 (7)
T2DM (%)	57 (11)	13 (39)	44 (9)
*HCV-MASLD*	236 (46)	22 (67)	214 (45)
*CMRF*			
Overweight or Obesity (%)	219 (43)	15 (45)	204 (43)
Prediabetes + T2DM (%)	311 (61)	21 (64)	290 (61)
Hypertension (%)	186 (36)	16 (48)	170 (35)
Hypertriglyceridemia (%)	113 (22)	13 (39)	100 (21)
Hypo-HDL cholesterolemia (%)	91 (18)	12 (36)	79 (16)
*Laboratory data at SVR24*			
AST, U/L	24 (18–27)	32 (22–34)	22 (18–26)
ALT, U/L	18 (12–20)	22 (14–25)	15 (12–20)
γ-GTP, U/L	18 (14–26)	20 (17–31)	18 (14–26)
Total bilirubin, mg/dL	0.7 (0.5–0.9)	0.8 (0.6–1.0)	0.7 (0.5–0.9)
Albumin, g/dL	4.3 (4.0–4.5)	4.1 (3.8–4.5)	4.3 (4.1–4.5)
Platelet count, × 10^4^/μL	17.0 (13.0–20.3)	12.5 (7.9–16.4)	16.6 (13.2–20.7)
FSG, mg/dL	112 (96–117)	125 (98–144)	104 (96–116)
HbA1c, %	6.1 (5.5–6.6)	6.0 (5.3–6.7)	5.9 (5.5–6.5)
Triglyceride, mg/dL	106 (72–151)	118 (69–149)	107 (72–151)
HDL-C, mg/dL	63 (50–76)	53 (46–62)	63 (51–76)
AFP, ng/mL	3.5 (2.1–4.5)	4.2 (3.1–5.0)	2.9 (2.0–4.4)
DCP, mAU/mL	17 (13–21)	16 (12–17)	17 (13–22)
*Fibroscan*			
CAP, dB/m	211 (173–247)	223 (194–263)	208 (172–245)
LSM, kPa	5.5 (4.1–9.0)	13.1 (6.5–16.3)	5.3 (4.0–8.7)
*Index and score*			
FIB-4 index	2.28 (1.54–3.31)	3.49 (2.38–6.59)	2.22 (1.52–3.26)
aMAP score	58 (54–65)	64 (60–68)	59 (54–64)
FAST score	0.13 (0.04–0.17)	0.26 (0.11–0.39)	0.08 (0.04–0.15)

Values are expressed as *n* (%) or median (first-third quartiles)

*AFP* alpha-fetoprotein, *ALT* alanine aminotransferase, *aMAP* age, male, albumin-bilirubin, platelets, *AST* aspartate aminotransferase, *BMI* body mass index, *CAP* Controlled Attenuation Parameter, *CMRF* cardiometabolic risk factors, *DCP* des-γ-carboxy prothrombin, *FAST* Fibro Scan-AST, *FIB-4* fibrosis-4, *FSG* fasting serum glucose, *HbA1c* hemoglobin A1c, *HCC* hepatocellular carcinoma, *HDL-C* high-density lipoprotein-cholesterol, *LSM* liver stiffness measurement, *MASLD* metabolic dysfunction-associated steatotic liver disease, *γ-GTP* γ-glutamyl transpeptidase, *T2DM* type 2 diabetes

### Factors associated with HCC development in patients who achieved SVR24

The clinical characteristics of patients who did and did not develop HCC were compared by univariate and multivariable analyses. Univariate analysis showed that T2DM, HCV-MASLD, hypertriglyceridemia, hypo-HDL cholesterolemia, AST, ALT, ɤ-GTP, albumin, platelet count, FSG, HDL-C, AFP, LSM, FIB-4 index, aMAP score, and FAST score were significantly associated with HCC development (Table 2). Multivariable analysis was performed for the remaining items that were significant on univariate analysis. Laboratory values included in the FIB-4 index, aMAP score, and FAST score were not included in the multivariable analysis to avoid confounding and to prevent multicollinearity. Since T2DM, hypertriglyceridemia, and hypo-HDL cholesterolemia are included in HCV-MASLD, multivariable analysis was conducted with HCV-MASLD, AFP, aMAP score, and FAST score. Both univariable and multivariable analyses were performed using the Fine and Gray subdistribution hazard model to account for the competing risk of de novo HCC. Multivariable analysis identified HCV-MASLD (SHR: 4.921, 95% CI: 1.899–12.75, *P* = 0.001), aMAP score (SHR: 1.106, 95% CI: 1.045–1.170, *P* < 0.001), and FAST score (SHR: 60.66, 95% CI: 8.676–424.1, *P* < 0.001) at the SVR24 time point as independent risk factors for the development of HCC after achieving SVR24. The predictive ability of each scoring system (FIB-4, aMAP, FAST) was evaluated using Harrell’s concordance index (c-index), which accounts for the time-to-event nature of HCC development. These values were calculated using Cox proportional hazards models to evaluate discriminative ability, while Fine and Gray models were used for primary risk analysis. The c-index was 0.750 (95% CI: 0.687–0.883) for FIB-4 index, 0.738 (95% CI: 0.687–0.883) for aMAP score, and 0.746 (95% CI: 0.687–0.883) for FAST score. All three scores demonstrated moderate to good discriminative performance for predicting HCC. These findings indicate that FIB-4, aMAP, and FAST scores all provide similarly useful information for risk stratification of post-SVR HCC.

**Table 2:** Risk factors of hepatocellular carcinoma occurrence in patients achieving sustained virological response

	Univariate analysis			Multivariable analysis		
	SHR	(95% CI)	*P*-value	SHR	(95% CI)	*P*-value
*Patient’s character*						
Age, years	1.029	(0.998–1.061)	0.067			
Gender, male/female	1.573	(0.799–3.098)	0.190			
BMI, kg/m^2^	0.994	(0.913–1.083)	0.890			
Smoker, yes/no	1.418	(0.718–2.802)	0.310			
Habitual alcohol, yes/no	2.254	(0.882–5.764)	0.090			
T2DM, yes/no	5.580	(2.806–11.10)	< 0.001			
*HCV-MASLD (with/without)*	5.045	(2.200–11.57)	< 0.001	4.921	(1.899–12.75)	0.001
*CMRF*						
Overweight or Obesity, yes/no	1.110	(0.565–2.182)	0.760			
Prediabetes + T2DM, yes/no	1.164	(0.575–2.357)	0.670			
Hypertension, yes/no	1.682	(0.849–3.331)	0.140			
Hypertriglyceridemia, yes/no	2.465	(1.232–4.932)	0.011			
Hypo-HDL cholesterolemia, yes/no	2.769	(1.372–5.589)	0.005			
*Laboratory data at SVR24*						
AST, U/L	5.359	(2.538–11.32)	< 0.001			
ALT, U/L	2.415	(1.306–4.466)	0.005			
γ-GTP, U/L	1.815	(1.201–2.745)	0.005			
Total bilirubin, mg/dL	1.298	(0.934–1.802	0.120			
Albumin, g/dL	0.256	(0.105–0.625)	0.003			
Platelet count, × 10^4^/μL	0.865	(0.805–0.930)	< 0.001			
FSG, mg/dL	1.010	(1.003–1.016)	0.003			
HbA1c, %	0.839	(0.448–1.571)	0.580			
Triglyceride, mg/dL	0.999	(0.996–1.002)	0.540			
HDL-C, mg/dL	0.962	(0.937–0.989)	0.006			
AFP, ng/mL	2.297	(1.156–4.566)	0.018	0.958	(0.795–1.154)	0.650
DCP, mAU/mL	0.536	(0.157–1.826)	0.320			
*Fibroscan*						
CAP, dB/m	1.005	(0.998–1.012)	0.130			
LSM, kPa	4.101	(2.369–7.100)	< 0.001			
*Index and score*						
FIB-4 index	1.302	(1.206–1.406)	< 0.001			
aMAP score	1.096	(1.057–1.136)	< 0.001	1.106	(1.045–1.170)	< 0.001
FAST score	66.13	(11.69–373.9)	< 0.001	60.66	(8.676–424.1)	< 0.001

*AFP* alpha-fetoprotein, *ALT* alanine aminotransferase, *aMAP* age, male, albumin-bilirubin, platelets, *AST* aspartate aminotransferase, *BMI* body mass index, *CAP* Controlled Attenuation Parameter, *CI* confidence interval, *CMRF* cardiometabolic risk factors, *DCP* des-γ-carboxy prothrombin, *FAST* Fibro Scan-AST, *FIB-4* fibrosis-4, *FSG* fasting serum glucose, *HbA1c* hemoglobin A1c, *HDL-C* high-density lipoprotein-cholesterol, *HR* hazard ratio, *LSM* liver stiffness measurement, *MASLD* metabolic dysfunction-associated steatotic liver disease, *γ-GTP* γ-glutamyl transpeptidase, *T2DM* type 2 diabetes

### HCC development rate according to the number of risk factors

The HCC development rate according to the number of risk factors, such as HCV-MASLD, aMAP score, and FAST score, was analyzed. aMAP score ≥ 61 or FAST score ≥ 0.12 were defined as HCC development risk factor based on optimal cutoffs derived from receiver operating characteristic (ROC) curve analysis. Patients with higher numbers of risk factors were more likely to develop HCC (Fig. 2b). The cumulative HCC development rates at 1, 3, 5, and 7 years were 1%, 4%, 7%, and 11% for patients who had 1 or 2 risk factors and 4%, 27%, 28%, and 46% for patients who had 3 risk factors, respectively (*P* < 0.001). Interestingly, only one of 141 patients who had no risk factors developed HCC approximately six years after SVR24. This result suggests that, even in the absence of risk factors, the risk of HCC development may re-emerge as the follow-up period extends. However, in the majority of cases, within five years after SVR24, HCC development was almost never observed in patients without risk factors.

### Relationships among the aMAP score, FAST score, and CMRFs

**Fig. 3:**
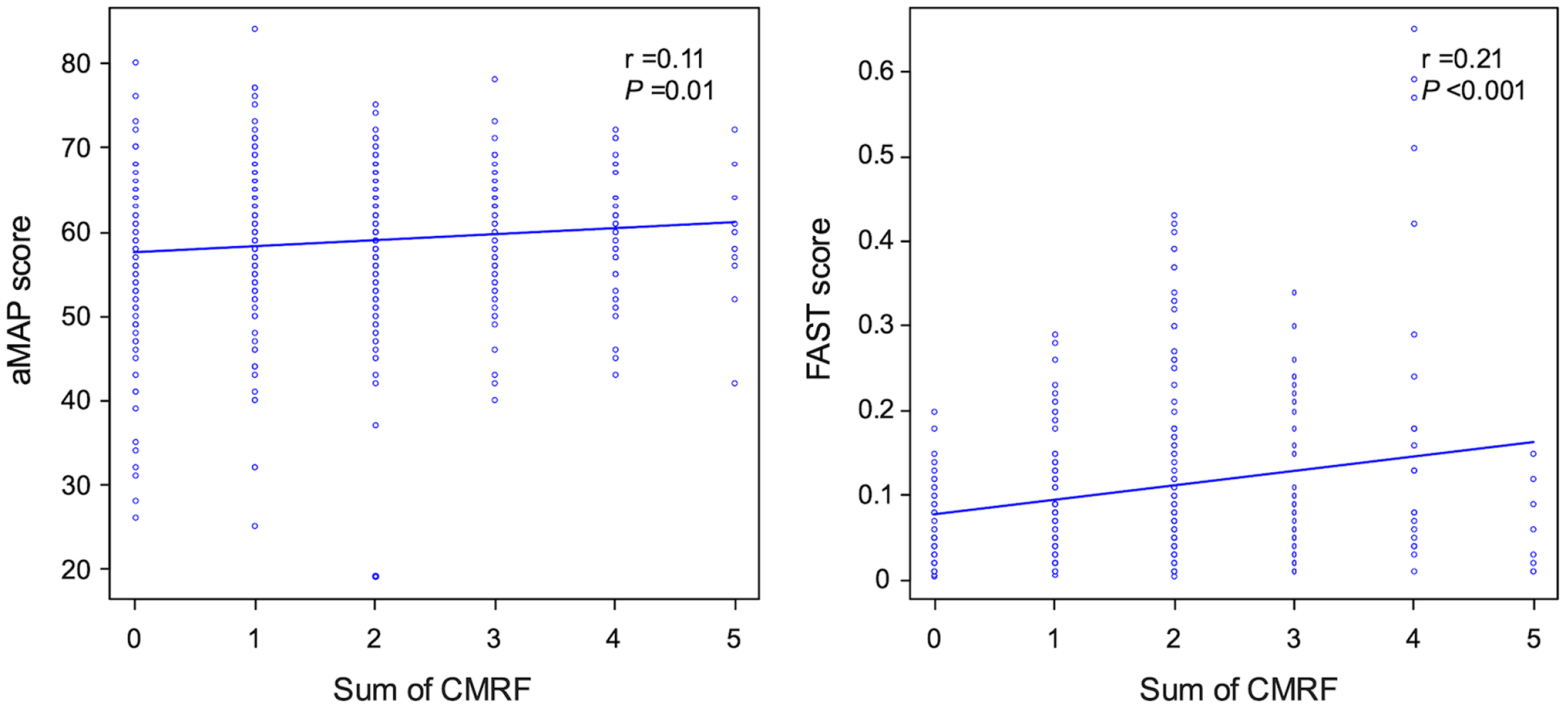
Correlation between the number of cardiometabolic risk factors (CMRFs) and aMAP and FAST scores was analyzed using Pearson's product-moment correlation coefficient. The number of CMRF was significantly correlated with both aMAP and FAST scores (*P* < 0.05 and *P* < 0.001, respectively)

The aMAP score and FAST score were identified as independent factors related to HCC development after SVR24. The correlations of the aMAP score, FAST score, and CMRFs were examined. Both the aMAP and Fast scores showed gradual positive correlations with an increasing number of CMRF items (*r* = 0.11, *P* = 0.01, and *r* = 0.21, *P* < 0.001, respectively) (Fig. 3). It was demonstrated that the presence of multiple CMRFs contributed to higher aMAP and FAST scores.

### Factors associated with HCC development after SVR24 in patients with MASLD

The risk factors for HCC development after HCV eradication were analyzed focused on patients with MASLD. The clinical characteristics of the 236 MASLD patients are shown in Table 3. Twenty-two of 236 MASLD patients (9.3%) developed HCC during the observation period. Continuous variables were analyzed as continuous variables without dichotomization to preserve statistical power. Serum markers with significantly skewed distributions, including AST, ALT, ɤ-GTP, total bilirubin, FSG, AFP, DCP and LSM, were log-transformed prior to analysis to improve distribution normality and model stability. Univariate analysis showed that hypertriglyceridemia, Hypo-HDL cholesterolemia, AST, platelet count, AFP, LSM, FIB-4 index, aMAP score, and FAST score were significantly associated with HCC development (Table 4). Although several variables showed significant associations with HCC occurrence in univariable analyses, only aMAP and FAST scores were included in the multivariable Fine–Gray model due to the limited number of events (*n* = 22) in order to avoid overfitting. Multivariable analysis identified aMAP score (SHR 1.085, *P* = 0.002) and FAST score (SHR 19.58, *P* = 0.005) as independent risk factors for HCC development after achieving SVR24 in MASLD patients.

**Table 3:** Characteristics of MASLD patients at SVR 24 weeks after the end of treatment in a combined cohort

	All patients		
	Combined cohort (*n* = 236)	HCC occurrence (*n* = 22)	HCC–free (*n* = 214)
*Patient’s character*			
Age, years	70 (60–77)	74 (68–81)	69 (59–76)
Gender, male (%)	88 (37)	8 (36)	80 (37)
BMI, kg/m^2^			
Smoker (%)	73 (31)	10 (45)	63 (29)
T2DM (%)	30 (13)	5 (23)	25 (12)
*CMRF*			
Overweight or Obesity (%)	97 (41)	11 (50)	86 (40)
Prediabetes + T2DM (%)	165 (70)	12 (55)	153 (71)
Hypertension (%)	105 (44)	11 (50)	94 (44)
Hypertriglyceridemia (%)	64 (27)	10 (45)	54 (25)
Hypo-HDL cholesterolemia (%)	53 (22)	9 (41)	44 (21)
*Laboratory data at SVR24*			
AST, U/L	22 (18–28)	27 (22–32)	22 (18–27)
ALT, U/L	14 (11–19)	18 (13–21)	14 (11–19)
γ–GTP, U/L	19 (14–26)	19 (16–27)	18 (14–25)
Total bilirubin, mg/dL	0.7 (0.6–0.9)	0.7 (0.6–0.9)	0.7 (0.5–0.9)
Albumin, g/dL	4.3 (4.0–4.5)	4.2 (4.0–4.5)	4.3 (4.0–4.5)
Platelet count, × 10^4^/μL	16.5 (12.7–20.7)	14.4 (9.7–16.8)	16.6 (13.0–21.4)
FSG, mg/dL	106 (98–121)	104 (96–121)	106 (99–120)
HbA1c, %	6.2 (5.6–6.7)	5.9 (5.5–6.3)	6.2 (5.6–6.8)
Triglyceride, mg/dL	108 (78–159)	100 (80–154)	108 (78–157)
HDL–C, mg/dL	58 (47–72)	51 (48–64)	58 (47–72)
AFP, ng/mL	3.1 (2.0–4.6)	4.0 (3.1–4.9)	2.9 (1.9–4.5)
DCP, mAU/mL	17 (13–22)	16 (12–17)	17 (13–23)
*Fibroscan*			
CAP, dB/m	220 (174–255)	221 (194–259)	220 (173–255)
LSM, kPa	5.4 (4.2–9.3)	9.6 (5.9–14.2)	5.3 (4.1–8.7)
*Index and score*			
FIB–4 index	2.53 (1.63–3.63)	3.39 (2.41–4.92)	2.39 (1.59–3.46)
aMAP score	60 (55–65)	65 (61–68)	60 (55–64)
FAST score	0.08 (0.04–0.17)	0.18 (0.11–0.34)	0.08 (0.04–0.14)

Values are expressed as *n* (%) or median (first–third quartiles)

*AFP* alpha–fetoprotein, *ALT* alanine aminotransferase, *aMAP* age, male, albumin–bilirubin, platelets, *AST* aspartate aminotransferase, *BMI* body mass index, *CAP* Controlled Attenuation Parameter, *CMRF* cardiometabolic risk factors, *DCP* des–γ–carboxy prothrombin, *FAST* Fibro Scan–AST, *FIB–4* fibrosis–4, *FSG* fasting serum glucose, *HbA1c* hemoglobin A1c, *HCC* hepatocellular carcinoma, *HDL–C* high–density lipoprotein–cholesterol, *LSM* liver stiffness measurement, *MASLD* metabolic dysfunction–associated steatotic liver disease, *γ–GTP* γ–glutamyl transpeptidase, *T2DM* type 2 diabetes

**Table 4:** Risk factors of hepatocellular carcinoma occurrence in MASLD patients achieving SVR24

	Univariate analysis			Multivariable analysis		
	SHR	(95% CI)	*P*-value	SHR	(95% CI)	*P*-value
*Patient’s character*						
Age, years	1.044	(0.999–1.091)	0.057			
Gender, male/female	1.072	(0.455–2.528)	0.870			
BMI, kg/m^2^	1.023	(0.944–1.108)	0.580			
Smoker, yes/no	1.838	(0.798–4.234)	0.150			
T2DM, yes/no	2.050	(0.791–5.318)	0.140			
*CMRF*						
Overweight or Obesity, yes/no	1.458	(0.639–3.326)	0.370			
Prediabetes + T2DM, yes/no	0.500	(0.219–1.141)	0.100			
Hypertension, yes/no	1.174	(0.512–2.694)	0.710			
Hypertriglyceridemia, yes/no	2.433	(1.052–5.628)	0.038			
Hypo-HDL cholesterolemia, yes/no	2.353	(1.009–5.485)	0.048			
*Laboratory data at SVR24*						
AST, U/L	3.609	(1.523–8.554)	0.004			
ALT, U/L	1.664	(0.839–3.222)	0.150			
γ–GTP, U/L	1.573	(0.872–2.839)	0.130			
Total bilirubin, mg/dL	1.097	(0.307–3.916)	0.890			
Albumin, g/dL	0.617	(0.227–1.673)	0.340			
Platelet count, × 10^4^/μL	0.875	(0.798–0.959)	0.004			
FSG, mg/dL	0.431	(0.035–5.322)	0.510			
HbA1c, %	0.553	(0.186–1.641)	0.290			
Triglyceride, mg/dL	0.977	(0.362–2.636)	0.960			
HDL–C, mg/dL	0.979	(0.943–1.016)	0.270			
AFP, ng/mL	2.356	(1.054–5.265)	0.037			
DCP, mAU/mL	0.415	(0.130–1.322)	0.140			
*Fibroscan*						
CAP, dB/m	1.004	(0.996–1.012)	0.370			
LSM, kPa	3.516	(1.720–7.187)	< 0.001			
*Index and score*						
FIB–4 index	1.293	(1.200–1.393)	< 0.001			
aMAP score	1.088	(1.041–1.137)	< 0.001	1.085	(1.028–1.145)	0.002
FAST score	21.67	(2.945–159.5)	0.003	19.58	(2.359–162.4)	0.005

*AFP* alpha–fetoprotein, *ALT* alanine aminotransferase, *aMAP* age, male, albumin–bilirubin, platelets, *AST* aspartate aminotransferase, *BMI* body mass index, *CAP* Controlled Attenuation Parameter, *CI* confidence interval, *CMRF* cardiometabolic risk factors, *DCP* des–γ–carboxy prothrombin, *FAST* Fibro Scan–AST, *FIB–4* fibrosis–4, *FSG* fasting serum glucose, *HbA1c* hemoglobin A1c, *HDL–C* high–density lipoprotein–cholesterol, *HR* hazard ratio, *LSM* liver stiffness measurement, *MASLD* metabolic dysfunction–associated steatotic liver disease, *γ–GTP* γ–glutamyl transpeptidase, *T2DM* type 2 diabetes

### Factors associated with HCC development after SVR in patients with non-MASLD

Finally, the risk factors for HCC development after HCV eradication in patients without MASLD (non-MASLD) were analyzed. The clinical characteristics of the 276 non-MASLD patients are shown in supplemental Table 1. Eleven of 276 non-MASLD patients (4.0%) developed HCC during the observation period. Univariate analysis showed that male, habitual alcohol, T2DM, AST, ALT, γ-GTP, albumin, platelet count, FSG, HDL-C, LSM, FIB-4 index, aMAP score, FAST score were significantly associated with HCC development (Supplemental Table 2). Because of the limited number of events (*n* = 11), T2DM, aMAP and FAST scores were included in the multivariable Fine–Gray model, T2DM (SHR 20.29, *P* < 0.001) and FAST score (SHR 575.9, *P* =  < 0.001) were identified as independent risk factors for HCC development after achieving SVR24 in non-MASLD patients.

## Discussion

The present study investigated the risk factors associated with HCC development in patients who achieved SVR following DAA therapy for HCV infection. HCV-MASLD, aMAP score, and FAST score were identified as independent risk factors for HCC development after SVR. Furthermore, a higher number of these risk factors was associated with an increased likelihood of HCC occurrence, reinforcing the importance of metabolic dysfunction in post-SVR liver carcinogenesis.

Historically, HCV infection was considered the predominant driver of hepatic carcinogenesis, primarily through persistent inflammation and fibrosis progression. However, with the advent of highly effective DAAs, viral eradication has substantially altered the landscape of post-SVR hepatic pathophysiology. Recent studies have emphasized the role of residual fibrosis and metabolic derangements as pivotal contributors to HCC development after SVR [8]. The present study corroborates these findings, underscoring the relevance of MASLD in HCC risk stratification.

The association between MASLD and HCC risk has been increasingly recognized, particularly in the context of metabolic dysfunction-driven hepatic injury. Recent nomenclature changes have introduced MASLD as an umbrella term replacing NAFLD, acknowledging the role of metabolic dysfunction beyond simple hepatic steatosis [10]. Studies have demonstrated that patients with MASLD have a significantly higher risk of HCC than those without MASLD [9]. The present study further supports this notion, showing that MASLD was an independent risk factor for HCC development post-SVR**.**

In the present study, aMAP and FAST scores were independently associated with HCC development risk, and both scores were also correlated with the number of CMRFs, suggesting that underlying metabolic dysfunction contributes to increased hepatic oncogenesis even after viral clearance. Sub-analysis showed that both aMAP and FAST scores were independently associated with the risk of HCC development in patients with MASLD, suggesting their potential utility as predictive markers in this subgroup. Moreover, the presence of T2DM and a high FAST score in non-MASLD patients at the time of SVR was also associated with de novo HCC development.

These findings suggest that metabolic dysfunction may play a critical role in hepatocarcinogenesis after SVR, not only in MASLD patients but also in non-MASLD patients with T2DM. Although these results are based on a limited number of events, incorporating aMAP and FAST scores into risk stratification in MASLD cases, and carefully monitoring T2DM status and FAST scores in non-MASLD patients, may help inform individualized HCC surveillance strategies.

Several studies have investigated the association between factors included as CMRFs and HCC. Obesity has been consistently associated with HCC risk [17]. Overweight promotes lipid peroxidation and excess free radical activity through SLD, increasing the risk of genomic mutations and potentially leading to HCC [18]. Moreover, adipose tissue secretes various adipokines, including tumor necrosis factor (TNF)-α, interleukin (IL)−6, leptin, and insulin-like growth factor (IGF-1), which are thought to have pathological effects in various cancers [19]. Some reports recognized the association between DM and HCC [17, 20]. In DM, hyperinsulinemia is observed, leading to an increase in IGF-1, which promotes the abnormal proliferation of hepatocytes. The elevation of IGF-1 is believed to alter cell cycle regulation and potentially increase the risk of HCC development [21, 22]. Several clinical studies showed that hypertension is a potential risk factor for liver injury and hepatic fibrosis [23]. In an animal model, Yoshiji et al. reported that angiotensin II progressed hepatic fibrosis in NAFLD rats, and the angiotensin-converting enzyme inhibitor decreased tumor growth by suppressing the endothelial vascular growth factor [24]. Opinions regarding the relationship between triglycerides and hepatocellular carcinoma vary, and no clear consensus has been reached. Some reports suggest a correlation between the TyG index and HCC development in patients with liver cirrhosis [25]. The protective effect of HDL-C against cancer appears to be associated with anti-inflammatory and anti-oxidative properties. HDL-C-associated apolipoproteins and adenosine triphosphate (ATP) binding cassette transporters are related to anti-tumor effects. HDL-C also has anti-apoptotic properties. Therefore, low HDL-C levels were associated with an increased risk of liver cancer compared with a moderate level of HDL-C [26]. We believe that therapeutic interventions for CMRFs, as well as lifestyle guidance including dietary and exercise therapy, can reduce the long-term risk of HCC. There is growing evidence that adherence to a healthy diet plays a role in delaying HCC development in at-risk populations. Epidemiological studies have suggested that increased consumption of fruits decreases the risk of HCC, and low vegetable intake was significantly associated with an increased risk of HCC [17]. An Italian case–control study reported an inverse relation between intakes of fruits, milk/yoghurt, white meats, and eggs, and HCC risk [17]. A prospective study observed a gradual correlation between decreased HCC risk and degree of physical activity [27]. Therefore, intervening in MASLD and CMRFs that develop after achieving SVR in chronic hepatitis C may similarly reduce the risk of HCC. Exploring this possibility will be the focus of our future research.

In the present study, 15 patients died during the follow-up period. Beyond hepatocarcinogenesis, MASLD has been increasingly recognized as a multisystem disease associated with a wide spectrum of extrahepatic complications. Recent studies have shown that MASLD significantly increases the risk of cardiovascular disease, including myocardial infarction, ischemic stroke, heart failure, and cardiovascular mortality. In particular, a large nationwide Korean cohort study demonstrated that MASLD was associated with a 1.39-fold higher risk of cardiovascular events, independent of traditional risk factors [28]. Additionally, a meta-analysis reported that MASLD is linked to a significantly higher risk of various cancers, including uterine, breast, prostate, colorectal, and lung cancers, with an overall incidence rate of 10.6 per 1000 person-years [29, 30]. Additionally, MASLD patients in the overlap group with MAFLD were found to have a higher risk of both all-cause and diabetes-related mortality [31]. Further prospective studies are needed to clarify the prognosis of MASLD patients after HCV eradication in the Japanese cohort.

These findings have significant clinical implications. The present results suggest that post-SVR patients with MASLD should also be considered for enhanced HCC monitoring in Japan. The stratification of post-SVR patients using aMAP and FAST scores could aid in identifying HCC high-risk individuals and optimizing surveillance strategies.

Despite its strengths, the present study has several limitations. First, it was a retrospective analysis conducted at a single center, which may limit the generalizability of the findings. Second, although non-invasive markers were used to assess liver fibrosis and steatosis, histological confirmation was not available in all cases. Third, SLD was evaluated only at the SVR24 time point. Since steatosis can change over time, the potential impact of temporal changes in SLD on HCC risk could not be assessed. Fourth, although hepatic steatosis is defined as CAP value of ≥ 248 dB/m (S1 grade) in EASL-EASD-EASO Clinical Practice Guidelines [32], only histological findings are consensus definition of SLD for the Japanese population. In this study, SLD was assessed using imaging modalities, but the optimal evaluation method for SLD remains an issue for future investigation. Future prospective studies incorporating serial imaging evaluations, liver biopsy, and long-term follow-up are warranted to clarify the dynamic role of steatosis and validate the present findings.

In conclusion, the present study focused on the importance of MASLD in relation to the risk of de novo HCC in chronic hepatitis C cases that achieved SVR24. This is the first report in Japan to describe hepatocarcinogenesis after hepatitis C treatment in relation to MASLD. The present study highlights the critical role of MASLD and metabolic dysfunction in post-SVR HCC risk. HCV-MASLD, aMAP score, and FAST score were identified as independent risk factors for HCC development, emphasizing the need for comprehensive metabolic assessment. Unlike previous reports primarily conducted in Taiwanese or Western populations, this study is the first to demonstrate the association between MASLD and de novo HCC development after HCV eradication in a Japanese cohort. This finding provides ethnically specific insights into post-SVR risk stratification and underscores the relevance of integrating MASLD assessment into long-term HCC surveillance.

## Supplementary Information

Below is the link to the electronic supplementary material.

Supplementary file1 (DOC 74 KB)

Supplementary file2 (DOC 98 KB)

## Abbreviations

AFP: Alpha-fetoprotein
aMAP: Age, male, albumin-bilirubin, platelets
Alb: Albumin
ALT: Alanine aminotransferase
AST: Aspartate aminotransferase
BMI: Body mass index
DAA: Direct-acting antiviral
CAP: Controlled attenuation parameter
CMRF: Cardiometabolic risk factors
c-index: Concordance index
CT: Computed tomography
DCP: Des-γ-carboxy prothrombin
DM: Diabetes mellitus
FAST: FibroScan-AST
FSG: Fasting serum glucose
HbA1c: Hemoglobin A1c
HCC: Hepatocellular carcinoma
HCV: Hepatitis C virus
HDL-C: High density lipoprotein-cholesterol
LSM: Liver stiffness measurement
MASLD: Metabolic dysfunction-associated steatotic liver disease
PT: Prothrombin time
SHR: Subdistribution hazard ratio
SLD: Steatotic liver disease
SVR: Sustained virologic response
T-Bil: Total bilirubin
T2DM: Type 2 diabetes mellitus
γ-GTP: γ-Glutamyl transpeptidase
