# Supplementary material for: Metabolic dysfunction-associated steatotic liver disease affects the development of hepatocellular carcinoma after sustained virologic response in chronic hepatitis C patients
Source: J Gastroenterol. 2025 Jul 7;60(8):1014–25. doi: 10.1007/s00535-025-02270-8 (PMC12289820; doi:10.1007/s00535-025-02270-8)
Supplement: Supplementary file 1 — Supplementary file1 (DOC 74 KB) [file 535_2025_2270_MOESM1_ESM.doc]

**Supplemental Table 1. Characteristics of non-MASLD** at SVR 24 weeks after the end of treatment in a combined cohort.

|  | All patients | | |
| --- | --- | --- | --- |
|  | Combined cohort  (n =276) | HCC occurrence  (n = 11) | HCC-free  (n = 265) |
| **Patient's character** |  |  |  |
| Age, years | 66 (56–74) | 62 (56–69) | 66 (56–74) |
| Gender, male (%) | 125 (45) | 9 (82) | 116 (44) |
| BMI, kg/m^2^ | 23 (20-25) | 22 (19-25) | 23 (20-25) |
| Smoker (%) | 116 (42) | 5 (45) | 111 (42) |
| Habitual alcohol (%) | 38 (14) | 5 (45) | 33 (12) |
| T2DM (%) | 27 (10) | 8 (73) | 19 (8) |
| **CMRF** |  |  |  |
| Overweight or Obesity (%) | 122 (44) | 4 (36) | 118 (45) |
| Prediabetes + T2DM (%) | 146 (53) | 9 (82) | 137 (52) |
| Hypertension (%) | 81 (29) | 5 (45) | 76 (29) |
| Hypertriglyceridemia (%) | 49 (18) | 3 (27) | 46 (17) |
| Low HDL-C (%) | 38 (14) | 3 (27) | 35 (13) |
| **Laboratory data at SVR24** |  |  |  |
| AST, U/L | 22 (18-26) | 36 (21-39) | 23 (20-25) |
| ALT, U/L | 16 (13-21) | 23 (18-26) | 16 (13-20) |
| γ-GTP, U/L | 18 (14-26) | 28 (21-37) | 18 (14-26) |
| Total bilirubin, mg/dL | 0.7 (0.5-0.9) | 0.9 (0.6-1.2) | 0.7 (0.5-0.9) |
| Albumin, g/dL | 4.3 (4.1-4.5) | 3.8 (3.7-4,3) | 4.3 (4.1-4.5) |
| Platelet count, ×10⁴/μL | 16.6 (13.2-19.9) | 11.3 (6.1-15.6) | 16.6 (13.3-20.1) |
| FSG , mg/dL | 102 (94-114) | 150 (119-159) | 102 (94-112) |
| HbA1c, % | 5.9 (5.4-6.4) | 6.3 (5.4-6.9) | 5.9 (5.4-6.4) |
| Triglyceride, mg/dL | 105 (65-144) | 102 (61-130) | 105 (66-144) |
| HDL-C, mg/dL | 65 (54-79) | 49 (41-60) | 68 (54-81) |
| AFP, ng/mL | 2.9 (2.2-4.2) | 3.6 (3.1-5.0) | 2.9 (2.2-4.2) |
| DCP, mAU/mL | 17 (14-21) | 15 (13-19) | 17(14-21) |
| **Fibroscan** |  |  |  |
| CAP, dB/m | 200 (171-233) | 212 (185-266) | 200 (171-232) |
| LSM, kPa | 5.6 (4.0-9.0) | 9.5 (6.8-29.1) | 5.3 (3.9-8.7) |
| **Index and score** |  |  |  |
| FIB-4 index | 2.17 (1.50–3.12) | 3.85 (2.39–7.79) | 2.16 (1.49–2.93) |
| aMAP score | 59 (53–64) | 66 (60–71) | 58 (53–64) |
| FAST score | 0.08 (0.04–0.16) | 0.30 (0.10–0.46) | 0.08 (0.04–0.16) |

Values are expressed as n (%) or median (first-third quartiles).

AFP, alpha-fetoprotein; ALT, alanine aminotransferase; aMAP, age, male, albumin-bilirubin, platelets; AST, aspartate aminotransferase; BMI, body mass index; CAP, Controlled Attenuation Parameter; CMRF, cardiometabolic risk factors; DCP, des-γ-carboxy prothrombin; FAST, Fibro Scan-AST; FIB-4, fibrosis-4; FSG, fasting serum glucose; HbA1c, hemoglobin A1c; HCC, hepatocellular carcinoma; HDL-C, high-density lipoprotein-cholesterol; LSM, liver stiffness measurement; MASLD, metabolic dysfunction-associated steatotic liver disease; γ-GTP, γ-glutamyl transpeptidase; T2DM, type 2 diabetes.
