# Supplementary material for: Metabolic dysfunction-associated steatotic liver disease affects the development of hepatocellular carcinoma after sustained virologic response in chronic hepatitis C patients
Source: J Gastroenterol. 2025 Jul 7;60(8):1014–25. doi: 10.1007/s00535-025-02270-8 (PMC12289820; doi:10.1007/s00535-025-02270-8)
Supplement: Supplementary file 2 — Supplementary file2 (DOC 98 KB) [file 535_2025_2270_MOESM2_ESM.doc]

**Supplemental Table 2. Risk factors of hepatocellular carcinoma occurrence in non-MASLD patients achieving SVR24**

|  | Univariate analysis | | |  | | | Multivariable analysis | | |
| --- | --- | --- | --- | --- | --- | --- | --- | --- | --- |
|  | SHR | (95% CI) | *P*-value | |  | SHR | | (95% CI) | *P*-value |
| **Patient's character** |  |  |  | |  |  | |  |  |
| Age, years | 0.992 | (0.959-1.027) | 0.660 | |  |  | |  |  |
| Gender, male / female | 5.617 | (1.198-26.34) | 0.029 | |  |  | |  |  |
| BMI, kg/m^2^ | 0.929 | (0.762-1.132) | 0.470 | |  |  | |  |  |
| Smoker, yes / no | 1.172 | (0.364-3.776) | 0.790 | |  |  | |  |  |
| Habitual alcohol, yes / no | 5.709 | (1.767-18.44) | 0.004 | |  |  | |  |  |
| T2DM, yes / no | 27.21 | (7.314-101.2) | <0.001 | |  | 20.29 | | (4.272-96.32) | <0.001 |
| **CMRF** |  |  |  | |  |  | |  |  |
| Overweight or Obesity, yes / no | 0.702 | (0.207-2.386) | 0.570 | |  |  | |  |  |
| Prediabetes+T2DM, yes / no | 3.990 | (0.871-18.28) | 0.075 | |  |  | |  |  |
| Hypertension, yes / no | 2.089 | (0.629-6.938) | 0.230 | |  |  | |  |  |
| Hypertriglyceridemia, yes / no | 1.741 | (0.482-6.286) | 0.400 | |  |  | |  |  |
| Hypo-HDL cholesterolemia, yes / no | 2.462 | (0.691-8.777) | 0.160 | |  |  | |  |  |
| **Laboratory data at SVR24** |  |  |  | |  |  | |  |  |
| AST, U/L | 8.662 | (3.153-23.79) | <0.001 | |  |  | |  |  |
| ALT, U/L | 5.077 | (2.067-12.47) | <0.001 | |  |  | |  |  |
| γ-GTP, U/L | 2.316 | (1.307-4.101) | 0.004 | |  |  | |  |  |
| Total bilirubin, mg/dL | 27.10 | (0.745-986.6) | 0.072 | |  |  | |  |  |
| Albumin, g/dL | 0.102 | (0.038-0275) | <0.001 | |  |  | |  |  |
| Platelet count, ×10⁴/μL | 0.837 | (0.736-0.952) | 0.007 | |  |  | |  |  |
| FSG, mg/dL | 33.60 | (8.374-134.8) | <0.001 | |  |  | |  |  |
| HbA1c, % | 1.377 | (0.462-4.105) | 0.570 | |  |  | |  |  |
| Triglyceride, mg/dL | 0.812 | (0.306-2.151) | 0.670 | |  |  | |  |  |
| HDL-C, mg/dL | 0.947 | (0.909-0.987) | 0.011 | |  |  | |  |  |
| AFP, ng/mL | 2.058 | (0.654-6.482) | 0.220 | |  |  | |  |  |
| DCP, mAU/mL | 0.912 | (0.270-3.075) | 0.880 | |  |  | |  |  |
| **Fibroscan** |  |  |  | |  |  | |  |  |
| CAP, dB/m | 1.004 | (0.991-1.018) | 0.510 | |  |  | |  |  |
| LSM, kPa | 5.594 | (2.200-14.22) | <0.001 | |  |  | |  |  |
| **Index and score** |  |  |  | |  |  | |  |  |
| FIB-4 index | 1.326 | (1.161-1.515) | <0.001 | |  |  | |  |  |
| aMAP score | 1.111 | (1.036-1.192) | 0.003 | |  | 1.071 | | (0.971-1.180) | 0.170 |
| FAST score | 308.1 | (23.53-4034) | <0.001 | |  | 575.9 | | (17.46-18990) | <0.001 |

AFP, alpha-fetoprotein; ALT, alanine aminotransferase; aMAP, age, male, albumin-bilirubin, platelets; AST, aspartate aminotransferase; BMI, body mass index; CAP, Controlled Attenuation Parameter; CI, confidence interval; CMRF, cardiometabolic risk factors; DCP, des-γ-carboxy prothrombin; FAST, Fibro Scan-AST; FIB-4, fibrosis-4; FSG, fasting serum glucose; HbA1c, hemoglobin A1c; HDL-C, high-density lipoprotein-cholesterol; HR, hazard ratio; LSM, liver stiffness measurement; MASLD, metabolic dysfunction-associated steatotic liver disease; γ-GTP, γ-glutamyl transpeptidase; T2DM, type 2 diabetes.
